# Dietary polyphenols in pediatric obesity and cardiometabolic risk: mechanisms and clinical evidence

**DOI:** 10.3389/fnut.2026.1849842

**Published:** 2026-06-05

**Authors:** Valeria Calcaterra, Hellas Cena, Maria Vittoria Conti, Ilaria Anna Maria Scavone, Sara Basilico, Antonia Quatrale, Gianvincenzo Zuccotti

**Affiliations:** 1 Department of Internal Medicine and Therapeutics, University of Pavia, Pavia, Italy; 2 Pediatric Department, Buzzi Children's Hospital, Milan, Italy; 3 Laboratory of Dietetics and Clinical Nutrition, Department of Public Health, Experimental and Forensic Medicine, University of Pavia, Pavia, Italy; 4 Clinical Nutrition Unit, ICS Maugeri IRCCS, Pavia, Italy; 5 Department of Biomedical and Clinical Science, University of Milano, Milan, Italy

**Keywords:** cardiometabolic risk, inflammation, insulin resistance, pediatric obesity, polyphenols

## Abstract

**Introduction:**

Pediatric obesity is a major global health concern and is associated with early cardiometabolic alterations, including insulin resistance, dyslipidemia, hypertension, endothelial dysfunction, and chronic low-grade inflammation. Dietary polyphenols, naturally present in fruits, vegetables, legumes, cocoa, and tea, have emerged as potential nutritional modulators of these pathways because of their antioxidant, anti-inflammatory, metabolic, and microbiota-related effects.

**Methods:**

This narrative overview summarizes current evidence on the role of dietary polyphenols in pediatric obesity and cardiometabolic risk. It examines the main biological mechanisms through which polyphenols may influence oxidative stress, inflammation, glucose and lipid metabolism, vascular function, and gut microbiota composition. It also reviews the available observational and clinical evidence in children and adolescents, with particular attention to polyphenol-rich dietary patterns.

**Results:**

Available evidence suggests that polyphenols may influence key mechanisms involved in pediatric obesity, including oxidative stress, inflammation, glucose and lipid metabolism, endothelial function, and gut microbiota balance. Observational studies indicate that polyphenol-rich dietary patterns, especially Mediterranean-style diets, are associated with more favorable adiposity and cardiometabolic profiles in children and adolescents. However, pediatric clinical trials remain limited and heterogeneous, preventing firm conclusions on efficacy and long-term safety.

**Conclusion:**

Polyphenols are biologically plausible contributors to cardiometabolic health in pediatric obesity, but current evidence mainly supports food-based dietary patterns rather than isolated supplementation. Larger pediatric-specific trials are needed to clarify their clinical relevance and long-term benefits.

## 1 Introduction

Pediatric obesity has reached epidemic proportions and is now recognized as one of the most pressing global public health challenges. According to estimates from the Global Burden of Disease 2021, the prevalence of overweight and obesity among children and adolescents more than doubled between 1990 and 2021, while obesity alone nearly tripled during the same period (1).

These findings are consistent with earlier global analyses showing a persistent rise in childhood obesity over recent decades (2). Of particular concern is the growing frequency of severe obesity across diverse settings, given its strong association with a higher burden of cardiometabolic complications (3).

Childhood and adolescent obesity is not simply a condition of excess adiposity, but a pathological state marked by early metabolic and vascular alterations. Longitudinal studies show that exposure to cardiometabolic risk factors early in life is associated with subclinical cardiovascular damage, including atherosclerosis and left ventricular hypertrophy, and with a greater likelihood of cardiovascular events by the fourth decade of life (4, 5). This risk is further amplified by adiposity tracking, whereby obesity arising early in life often persists into adolescence and adulthood, prolonging exposure to hyperglycemia, dyslipidemia, and hypertension (6). As a result, complications may develop earlier and contribute to a greater cumulative burden of vascular and metabolic damage across the life course (7).

In the pediatric population, cardiometabolic risk is currently conceptualized as a cluster of interconnected abnormalities, encompassing visceral obesity, insulin resistance and dysglycemia, atherogenic dyslipidemia (characterized by hypertriglyceridemia and reduced HDL-C levels), and arterial hypertension (8). Although diagnostic criteria remain heterogeneous, there is broad agreement that these four components constitute the core features of the pediatric metabolic syndrome, whose prevalence among children and adolescents with obesity ranges from 6 to 39% (9).

From a pathophysiological perspective, insulin resistance represents the central mechanistic hub integrating visceral adiposity, dyslipidemia, and hypertension. Expansion of visceral adipose tissue is accompanied by a state of low-grade chronic inflammation, increased circulating free fatty acids, and altered adipocytokine secretion, all of which contribute to impaired insulin sensitivity in hepatic and skeletal muscle tissues. These mechanisms collectively promote hypertriglyceridemia, reduced HDL-C levels, and increased production of small, dense LDL particles, thereby driving the atherogenic dyslipidemia characteristic of obesity (10, 11).

In parallel, compensatory hyperinsulinemia and adipocyte dysfunction contribute to the activation of both the sympathetic nervous system and the renin–angiotensin–aldosterone system, thereby promoting sodium retention, increased peripheral vascular resistance, and the development of hypertension (12). Collectively, these mechanisms lead to early endothelial dysfunction and accelerate cardiovascular damage beginning in childhood.

In this context, there is growing interest in “biologically targeted” nutritional strategies capable of modulating shared inflammatory and metabolic pathways. Dietary polyphenols, abundantly present in fruits, vegetables, cocoa, tea, and legumes, have been proposed as biologically plausible modulators of cardiometabolic dysfunction. They influence redox balance, inflammatory signaling pathways, endothelial function, and lipid and glucose metabolism, as well as the composition of the gut microbiota, all of which are central to the pathogenesis of obesity-related metabolic complications (13).

Human nutrigenomic studies suggest that these compounds may influence gene expression and epigenetic regulation of key metabolic pathways, particularly those involved in insulin sensitivity and lipid homeostasis (14, 15). Although most intervention studies have been conducted in adults, emerging evidence indicates that, even during adolescence, adherence to dietary patterns rich in plant-derived bioactive compounds is associated with a more favorable metabolic profile, including improved blood pressure and lipid parameters (16). The early appearance of oxidative stress and endothelial dysfunction in childhood obesity further suggests that specific developmental windows may be especially responsive to nutritional interventions (17). This concept is supported by the greater metabolic plasticity that characterizes childhood and adolescence, implying that interventions introduced at earlier stages may produce greater benefits than those implemented later in life (17).

Moreover, the gut microbiota, particularly dynamic during childhood, may act as an important mediator of polyphenol effects through the production of bioactive metabolites capable of modulating inflammation and insulin sensitivity (18).

Nevertheless, several issues remain unresolved, including bioavailability, optimal dosing, the influence of pubertal stage and gut microbiota composition, and the long-term effectiveness of these compounds in pediatric populations (19–21).

Against this background, this narrative review provides a critical and up-to-date overview of the evidence on the role of dietary polyphenols in pediatric obesity and cardiometabolic risk. It summarizes the main biological mechanisms involved, including oxidative stress, chronic low-grade inflammation, insulin resistance, dyslipidemia, endothelial dysfunction, and gut microbiota interactions, and examines the available clinical and observational evidence in children and adolescents. By integrating mechanistic and clinical findings, this review highlights the potential relevance of polyphenol-rich dietary patterns for early-life cardiometabolic health and identifies key gaps for future research.

## 2 Dietary polyphenols: classification and sources in the pediatric diet

### 2.1 Main classes of dietary polyphenols

Dietary polyphenols are a large and chemically diverse group of plant secondary metabolites characterized by the presence of one or more aromatic rings bearing hydroxyl substituents (22). To date, more than 8,000 individual phenolic compounds have been identified across medicinal and edible plants, making polyphenols the most abundant antioxidants in the human diet (22). They are widely distributed in vegetables, fruits, cereals, legumes, nuts, and plant-derived beverages such as tea, coffee, and red wine (23). Based on their chemical structure, specifically the number of phenol rings and the structural elements that link them, dietary polyphenols are generally classified into four main groups: flavonoids, phenolic acids, stilbenes, and lignans, with additional subclasses such as tannins sometimes considered separately (22, 24). While this classification is structurally driven, it is also relevant from a nutritional perspective, as different classes show distinct bioavailability, metabolism, and dietary distribution patterns.

Flavonoids represent the largest and most extensively studied class of dietary polyphenols. They share a common C6–C3–C6 structure and are further subdivided into several subclasses, including flavanols, flavonols, flavones, flavanones, isoflavones, and anthocyanins (25). These compounds are widely distributed in plant-based foods, with tea, cocoa, fruits, vegetables, and legumes representing major dietary sources. Rather than individual compounds, flavonoids are typically consumed as complex mixtures, predominantly in glycosylated or conjugated forms, which strongly influence their absorption, bioavailability, and metabolic fate (24). Notably, subclasses such as flavanols and anthocyanins are among the most abundant flavonoids in the diet and are largely responsible for the polyphenol content of commonly consumed foods such as tea, cocoa, and berries.

Phenolic acids constitute another major class of dietary polyphenols and are commonly divided into hydroxybenzoic and hydroxycinnamic acids. Among these, hydroxycinnamic acids, including caffeic, ferulic, p-coumaric, and chlorogenic acids, are quantitatively the most relevant in the human diet and are widely present in coffee, whole grains, fruits, and vegetables (26). A distinctive feature of phenolic acids is that they are often bound to the plant cell wall matrix, particularly in cereals, which influences their release during digestion and contributes to their relatively low bioavailability compared with other polyphenol classes (25).

Stilbenes represent a smaller group of dietary polyphenols, and they are typically produced by plants in response to stress conditions such as pathogen attack or UV radiation (25, 26). The most widely known stilbene is resveratrol, which is present in grapes, red wine, peanuts, and certain berries (27).

Lignans are formed by the dimerization of phenylpropanoid units and are widely distributed in plant-based foods, particularly seeds, whole grains, and legumes (28). Flaxseeds and sesame seeds represent the richest dietary sources of lignans (22, 29). Other relevant sources include whole-grain cereals, berries, and certain vegetables such as broccoli and carrots (24, 30).

In addition to these major classes, polyphenols also include complex compounds such as tannins, which can be subdivided into hydrolysable tannins and condensed tannins (proanthocyanidins). These compounds are abundant in foods such as tea, cocoa, grapes, and berries and often contribute substantially to total polyphenol intake, particularly through commonly consumed beverages (26, 30).

Overall, the diversity of polyphenols in plant foods reflects the wide range of botanical sources and biosynthetic pathways involved in their production (25, 29). Fruits, vegetables, whole grains, legumes, tea, coffee, cocoa, and plant-derived beverages collectively represent the primary contributors to dietary polyphenol intake in human populations (22, 25, 29).

### 2.2 Food sources commonly consumed by children

While the general landscape of polyphenol-rich foods in adults has been extensively characterized using databases such as Phenol-Explorer (31) and the USDA Flavonoid Database (32), population-level data specifically focused on children and adolescents remain relatively limited (22, 33). Nevertheless, several cross-sectional studies conducted in European and other Western cohorts have begun to map the major dietary contributors to polyphenol intake in younger age groups, revealing patterns that differ substantially from those observed in adults (34, 35).

Fruits consistently emerge as the leading polyphenol source in children and adolescents. Data from the HELENA study (34), a large multicenter cross-sectional study of 2,428 European adolescents, identified fruit as the single largest contributor to total polyphenol intake, accounting for approximately 23% of total intake, with apples and pears alone representing 16.3% (34). This finding is consistent with data from the UK National Diet and Nutrition Survey Rolling Programme (NDNS RP, 2008–2014) (35), which analyzed 4,636 children aged 1.5–18 years and found flavan-3-ols and hydroxycinnamic acids to be the most consumed polyphenol classes across all pediatric age group (35). Apples are of particular interest given their content of flavanol monomers (epicatechin), procyanidin oligomers (especially procyanidin B2), chlorogenic acid, and quercetin glycosides concentrated in the skin (23, 24). Berries, including strawberries, blueberries, raspberries, and blackberries, are rich sources of anthocyanins, ellagitannins, and phenolic acids and, when consumed, contribute substantially to the flavonoid fraction of children's diets (24, 36). Citrus fruits, such as oranges, mandarins, and lemons, provide flavanones (hesperetin and naringenin) that are characteristic of this fruit family and frequently enter children's diets through fruit juices or juice-based beverages (34, 37).

Vegetables provide a range of polyphenols across subclasses, though their contribution to overall pediatric polyphenol intake tends to be lower than that of fruit, partly reflecting lower vegetable consumption in this age group, estimated at approximately half of the recommended amount in European adolescents (38). Onions are particularly rich in quercetin glycosides; tomatoes provide chlorogenic acid and naringenin; broccoli and other cruciferous vegetables contribute hydroxycinnamic acids; and spinach supplies flavones such as luteolin and apigenin (22, 34).

Fruit and vegetable juices constitute a particularly relevant vehicle of polyphenol exposure in children, given their wide acceptance and frequent consumption in this age group (39, 40). In the HELENA study, fruit and vegetable juices accounted for 15.6% of total polyphenol intake in European adolescents (34). However, two main concerns arise regarding the consumption of fruit juices. First, even when labeled as 100% fruit or vegetable juice, these beverages contain high amounts of free sugars and lack the dietary fiber present in whole fruits and vegetables, which may facilitate excessive intake (41, 42). Second, commercial juice processing, including clarification, filtration, and pasteurization, can significantly reduce flavonoid concentrations, meaning that commercially produced juices generally contain lower levels of polyphenols compared with their whole fruit counterparts (24).

Legumes are valuable sources of flavonoids, particularly isoflavones (in soy) and condensed tannins, as well as hydroxycinnamic acids (22, 24). Despite their nutritional value, legume consumption among children in many Western countries remains below recommendations (43, 44).

Cereals and cereal-based foods commonly consumed during childhood, such as whole wheat bread, oats, barley, and brown rice, also contribute to polyphenol intake, such as phenolic acids, particularly ferulic acid and p-coumaric acid (24, 45). These compounds are largely bound to the cereal fiber matrix and are concentrated in the bran layer, explaining the markedly higher polyphenol content of whole grain products compared with refined cereals (22, 29). Potatoes, widely consumed in pediatric diets, also represent a notable source of hydroxycinnamic acids, particularly chlorogenic acid, and may contribute substantially to overall polyphenol intake despite not being traditionally considered a polyphenol-rich vegetable (34, 35).

Cocoa and cocoa-derived foods, including chocolate and cocoa beverages, represent a relevant source of polyphenols in children's diets, accounting for approximately 19.2% of total polyphenol intake in the HELENA study (34). Cocoa is one of the richest dietary sources of flavanols, particularly epicatechin, catechin, and procyanidins (29, 36). Among cocoa-derived products, dark chocolate with a high cocoa content (≥70%) generally retains higher concentrations of flavanols, whereas milk chocolate undergoes substantial losses due to alkaline processing and dilution (22, 24). However, it should be acknowledged that the recommended serving size of chocolate and chocolate-based products is generally small due to the presence of other components, such as sugars and saturated fats, which limits their overall contribution to polyphenol intake.

Other non-alcoholic beverages deserve specific mention in the context of pediatric polyphenol intake. Whilst tea and coffee, the dominant polyphenol sources in adults across multiple European populations, are rarely consumed by young children, their intake increases during adolescence (34, 35). In the NDNS RP cohort, non-alcoholic beverages including tea were already among the leading contributors to polyphenol intake in older children and teenagers (35).

It is also important to consider that many foods consumed by children undergo varying degrees of industrial processing, which may influence the final polyphenol content through degradation, dilution, or removal of phenolic-rich fractions during refining and manufacturing processes (24, 29). Overall, dietary polyphenol intake in children largely reflects the consumption of commonly available plant-based foods within daily meals and snacks. Fruits, vegetables, whole grains, cocoa products, and plant-derived beverages therefore represent the principal contributors to polyphenol exposure in pediatric diets, although their relative importance varies according to age, cultural dietary patterns, and food availability.

The main dietary sources of polyphenols commonly consumed in pediatric populations, along with their predominant polyphenol classes, are summarized in Table 1.

**Table 1:** Main dietary sources of polyphenols commonly consumed in pediatric populations.

Food source	Main polyphenol classes	Examples of compounds	Approximate content
Apples	Flavonoids, phenolic acids	Quercetin, chlorogenic acid, and catechin	Moderate
Berries (strawberries, blueberries)	Anthocyanins, ellagitannins	Cyanidin, delphinidin	High
Citrus fruits	Flavanones	Hesperidin, naringenin	Moderate
Vegetables (onions, broccoli, and spinach)	Flavonols, hydroxycinnamic acids	Quercetin, luteolin	Moderate
Whole grains	Phenolic acids	Ferulic acid, p-coumaric acid	Moderate
Legumes	Flavonoids, tannins	Isoflavones, proantocianidins	Moderate
Cocoa products	Flavanols	Epicatechin, catechin	High
Fruit juices	Flavonoids, phenolic acids	Various	Variable

### 2.3 Dietary patterns rich in polyphenols

As polyphenols are predominantly found in plant-based foods, dietary patterns characterized by a high consumption of fruits, vegetables, whole grains, legumes, nuts, and plant-derived beverages tend to provide the greatest amounts and diversity of dietary polyphenols (24, 26). In contrast, dietary patterns dominated by refined grains, ultra-processed foods, and sugar-sweetened beverages typically result in substantially lower polyphenol exposure.

The Mediterranean diet (MD) is widely recognized as one of the richest dietary patterns in polyphenols due to its emphasis on fruits and vegetables, often consumed seasonally, as well as legumes, whole grains, nuts, extra-virgin olive oil, and a limited intake of red and processed meat (46). In the PREDIMED (PREvención con DIeta MEDiterránea) trial, a large randomized, multicenter, controlled 5-year feeding study involving 7,200 participants at high cardiovascular risk, mean total polyphenol intake was 820 ± 323 mg/day, comprising 443 ± 218 mg/day of flavonoids and 304 ± 156 mg/day of phenolic acids, calculated using the Phenol-Explorer database (47). A specialized variant of this dietary model, known as the Green-MED diet, has been developed to further increase polyphenol intake while reducing red meat consumption (48). In clinical trials such as DIRECT-PLUS, this pattern included the daily consumption of walnuts, providing approximately 440 mg/day of polyphenols, as well as 3–4 cups of green tea and a Wolffia globosa-based green shake, contributing an additional 800 mg/day of polyphenols (48). This variant was specifically designed to maximize dietary exposure to polyphenol-rich foods.

In addition to the Mediterranean dietary pattern, several other dietary models characterized by high plant food consumption also provide substantial amounts of polyphenols. The Dietary Approaches to Stop Hypertension (DASH) diet, originally developed to reduce blood pressure, promotes the intake of fruits, vegetables, whole grains, legumes, nuts, and low-fat dairy products, while limiting sodium, saturated fat, and refined sugars (49). Although not specifically designed as a polyphenol-rich diet, its structural emphasis on plant-based foods results in a high intake of polyphenols, including flavonols (e.g., quercetin from vegetables such as onions and broccoli), flavanones (hesperetin and naringenin from citrus fruits), and hydroxycinnamic acids, as well as additional contributions from whole grains, legumes, and nuts (49, 50). The Mediterranean-DASH Intervention for Neurodegenerative Delay (MIND) diet combines elements of both the Mediterranean and DASH dietary patterns (51). It emphasizes the consumption of polyphenol-rich foods such as berries, leafy green vegetables, olive oil, and tea, further supporting a high dietary intake of diverse polyphenolic compounds (52). Similarly, the Nordic diet (53), characterized by high consumption of whole grains, root vegetables, legumes, berries, and fish, represents another dietary model associated with elevated polyphenol intake due to its reliance on plant-based foods typical of Northern European regions (54, 55). In the same way, traditional Japanese dietary patterns also provide substantial amounts of polyphenols, largely due to the habitual consumption of tea, soy-based products (including tofu, miso, and natto), and a wide variety of plant-derived foods (56, 57). These dietary habits contribute a diverse range of polyphenol subclasses, including isoflavones, flavonoids, and phenolic acids (56, 57). Finally, plant-based dietary patterns, ranging from vegetarian to strictly vegan diets, are inherently rich in polyphenols due to their predominant reliance on plant-derived foods. Evidence suggests that these dietary patterns provide higher polyphenol intakes compared with typical Western diets, which are generally characterized by lower consumption of fruits, vegetables, and whole plant foods (58). Overall, dietary patterns rich in plant-based foods represent the primary drivers of polyphenol intake and are commonly used as practical proxies for polyphenol exposure in epidemiological research. This approach is particularly relevant in pediatric populations, where direct and standardized measures of polyphenol intake are still scarce. The quantity and diversity of polyphenols therefore largely depend on the composition and quality of the overall diet, with variations across populations driven by age, cultural dietary habits, and food availability.

## 3 Relevant biological mechanisms of dietary polyphenols

### 3.1 Antioxidant activity and reduction of oxidative stress

Oxidative stress is a key mechanism linking obesity to cardiometabolic complications. Antioxidant properties are a common feature of most dietary polyphenols, although their magnitude and underlying mechanisms may vary depending on chemical structure, bioavailability, and metabolic transformation. Excess adiposity is characterized by several cellular alterations, including mitochondrial dysfunction, increased production of reactive oxygen species (ROS), and activation of cellular stress pathways, ultimately leading to tissue damage and metabolic dysregulation that promote insulin resistance and hepatic steatosis (59, 60). At the cellular level, ROS accumulation induces oxidative damage to lipids, proteins, and DNA, promoting lipid peroxidation and impairing mitochondrial function (61). Adipose tissue, in particular, represents a major source of ROS in obesity and is characterized by the activation of redox-dependent inflammatory pathways, which drive systemic metabolic deterioration (62).

Dietary polyphenols exhibit antioxidant properties through both direct and indirect mechanisms. They act as direct scavengers of free radicals and, in parallel, modulate intracellular signaling pathways involved in redox homeostasis (63).

Their scavenging activity relies on the ability to donate a hydrogen atom or an electron to unstable radical species, thereby stabilizing the resulting radical. The main mechanisms described include hydrogen atom transfer (HAT), single-electron transfer (SET), and transition metal chelation, which limits the formation of highly reactive radicals catalyzed by iron and copper (61, 64, 65).

However, direct radical scavenging alone does not fully explain the biological effects of polyphenols *in vivo*, as their systemic bioavailability is often limited and plasma concentrations achieved after dietary intake are typically lower than those used in *in vitro* systems.

Indeed, the circulating concentrations of polyphenols and their metabolites after dietary intake are generally much lower than those required to directly neutralize reactive oxygen species in a stoichiometric manner. Conversely, even low concentrations are sufficient to interact with molecular targets involved in redox-sensitive signaling pathways, supporting the concept that polyphenols primarily act as modulators of cellular signaling rather than as direct antioxidants *in vivo* (61, 65).

Accordingly, growing evidence indicates that their effects are largely mediated by modulation of cell signaling and gene expression rather than by stoichiometric radical neutralization (61, 65).

Polyphenols have been shown to activate the Keap1–Nrf2/ARE pathway, a major cellular antioxidant defense system, thereby promoting the expression of detoxifying and antioxidant enzymes such as superoxide dismutase, catalase, and glutathione peroxidase (66–68).

Nrf2 also interacts with key metabolic signaling nodes, including AMPK, PI3K/Akt, and NF-κB, highlighting the close interplay between redox regulation, inflammation, and energy metabolism (59, 69).

In addition to Nrf2 activation, polyphenols modulate other redox-sensitive pathways. They exert inhibitory effects on NF-κB and MAPKs, leading to reduced transcription of pro-inflammatory and pro-oxidant genes (70). Moreover, they inhibit pro-oxidant enzymes, including xanthine oxidase, and chelate redox-active transition metals, further reducing intracellular oxidative stress (71, 72).

Among the various polyphenols, curcuminoids from Curcuma longa have been extensively studied as representative compounds with well-characterized antioxidant and metabolic effects. Curcumin is presented here as a representative example, although similar antioxidant and signaling effects have been described for a wide range of dietary polyphenols. It reduces ROS production, improves mitochondrial function, and modulates multiple redox-sensitive pathways (73).

Experimental studies suggest that this compound ameliorates obesity-associated metabolic alterations by reducing ROS accumulation in adipose tissue and liver, thereby contributing to improved insulin sensitivity and lipid metabolism (74).

Similarly, Nrf2- and AMPK-mediated antioxidant and cytoprotective effects, along with modulation of inflammatory pathways, have been reported for resveratrol, quercetin, catechins, oleuropein, and hydroxytyrosol. Although these compounds share common mechanistic features, differences in chemical structure, bioavailability, and metabolism account for their distinct biological effects (59, 75, 76).

The antioxidant potential of polyphenols should not be interpreted solely as a direct scavenging action. Rather, they act as integrated modulators of the cellular redox response, combining radical neutralization, metal chelation, inhibition of pro-oxidant enzymes, and activation of cytoprotective pathways. These coordinated mechanisms may contribute to the prevention of cardiometabolic complications associated with obesity, including in pediatric populations.

### 3.2 Modulation of low-grade chronic inflammation

Low-grade chronic inflammation is a hallmark of obesity and is closely linked to metabolic dysfunction (77). Adipose tissue expansion promotes increased secretion of pro-inflammatory cytokines, contributing to a persistent inflammatory microenvironment that disrupts metabolic homeostasis (78, 79).

Importantly, adipose tissue is a complex and heterogeneous organ composed not only of adipocytes but also of various non-adipocyte cell types, including immune cells (such as macrophages), endothelial cells, and fibroblasts. Both adipocytes and cells of the stromal vascular fraction contribute to the production of pro-inflammatory mediators, collectively shaping the inflammatory microenvironment associated with obesity (78, 79).

Within this context, a key event in obesity-induced inflammation is the accumulation of macrophages in adipose tissue, predominantly of the pro-inflammatory M1 phenotype, resulting in increased production of cytokines such as tumor necrosis factor-α (TNF-α), interleukin-6 (IL-6), and C-reactive protein (CRP), which impair metabolic signaling pathways (78–80).

At the molecular level, these cytokines activate critical intracellular pathways, including c-Jun N-terminal kinase (JNK) and IκB kinase β (IKKβ), thereby impairing insulin signaling and promoting insulin resistance (81, 82).

Activation of innate immunity receptors, particularly Toll-like receptor 4 (TLR4), by saturated fatty acids and lipopolysaccharides (LPS) further amplifies inflammation through NF-κB and MAPK activation (83). In parallel, activation of the NLRP3 inflammasome promotes the maturation and release of IL-1β and IL-18, further exacerbating metabolic inflammation and insulin resistance (84, 85).

Polyphenols exert anti-inflammatory effects primarily by modulating key intracellular pathways, notably NF-κB, a central transcriptional regulator linking inflammation to metabolic dysfunction (86, 87).

Through NF-κB inhibition, polyphenols reduce the expression of pro-inflammatory cytokines and adhesion molecules. In addition, they modulate MAPK, JNK, and IKKβ pathways, thereby contributing to the restoration of insulin signaling and attenuation of systemic inflammation. Certain polyphenols also attenuate TLR4 activation and NLRP3 inflammasome activity, suggesting a broader regulatory effect of innate immunity and metabolic inflammation (79, 86, 88).

Polyphenols further influence immune function within adipose tissue by modulating macrophage polarization, promoting a shift from the pro-inflammatory M1 phenotype to the anti-inflammatory M2 phenotype, thereby contributing to the resolution of metabolic inflammation (89, 90).

Another important mechanism involves the modulation of adipokines. Adiponectin, an anti-inflammatory and insulin-sensitizing hormone reduced in obesity, exerts beneficial effects partly through AMPK activation and inhibition of NF-κB signaling (91).

Clinical and experimental evidence indicates that polyphenols may increase adiponectin levels and modulate adipokine profiles, thus contributing to improved metabolic homeostasis and reduced cardiometabolic risk (92–95).

Therefore, these findings indicate that polyphenols act on multiple inflammatory pathways associated with obesity, including NF-κB, JNK, IKKβ, TLR4, and NLRP3, thereby helping restore immunometabolic balance and prevent metabolic complications.

### 3.3 Effects on glucose metabolism and insulin sensitivity

Altered glucose metabolism and reduced insulin sensitivity are central metabolic disturbances associated with pediatric obesity. Excess adiposity and chronic low-grade inflammation contribute to insulin resistance by activating inflammatory and metabolic pathways that interfere with insulin signaling (79).

At the molecular level, insulin resistance is characterized by impaired activation of the PI3K/Akt pathway, which regulates GLUT4 translocation to the plasma membrane and subsequent glucose uptake (96, 97).

Polyphenols influence glucose homeostasis through multiple mechanisms involving both inflammatory and metabolic pathways. They modulate intracellular signaling pathways associated with insulin action, enhancing insulin sensitivity and promoting GLUT4 translocation in peripheral tissues, thereby restoring glucose uptake (98, 99).

AMP-activated protein kinase (AMPK), a cellular energy sensor, plays a central role in this process. AMPK activation promotes glucose uptake, enhances fatty acid oxidation, and inhibits hepatic gluconeogenesis, collectively improving insulin sensitivity and metabolic homeostasis (100).

Polyphenols are widely described as AMPK activators, suggesting that this pathway mediates many of their metabolic effects. Additionally, polyphenols modulate other signaling pathways, including PI3K/Akt and MAPK, contributing to improved metabolic function and attenuation of obesity-associated inflammation (101, 102), and may also influence cAMP response element-binding protein (CREB), a transcription factor involved in the regulation of genes controlling glucose metabolism, including gluconeogenesis and insulin signaling. Through modulation of CREB activity, polyphenols may contribute to the regulation of hepatic glucose production and overall metabolic homeostasis (99).

### 3.4 Effects on lipid metabolism and endothelial function

Altered lipid metabolism and endothelial dysfunction are major contributors to obesity-related cardiometabolic risk. Excess adiposity is associated with an atherogenic lipid profile, while chronic inflammation and oxidative stress impair nitric oxide bioavailability and promote early vascular damage (103).

At the molecular level, endothelial dysfunction in obesity is linked to reduced activity of endothelial nitric oxide synthase (eNOS) and increased ROS production, which inactivate NO and promote a pro-oxidant and pro-inflammatory vascular environment. Activation of inflammatory pathways, including NF-κB, further contributes to the expression of adhesion molecules and vascular dysfunction (104–106).

Polyphenols modulate lipid metabolism through multiple mechanisms. They regulate transcription factors involved in lipid homeostasis, including peroxisome proliferator-activated receptors (PPARα and PPARγ) and sterol regulatory element-binding proteins (SREBP-1c), thereby promoting fatty acid oxidation and reducing hepatic lipogenesis, ultimately improving lipid profiles and reducing ectopic lipid accumulation (99, 107, 108).

In vascular tissues, polyphenols enhance endothelial function by increasing NO bioavailability, inhibiting pro-oxidant enzymes, and reducing expression of endothelial inflammatory mediators (103).

Dietary patterns rich in plant-derived bioactive compounds have been associated with improved lipid profiles and reduced hepatic fat accumulation (60), partly attributable to the anti-inflammatory and antioxidant properties of polyphenols (109). Additionally, polyphenols have been shown to improve cardiovascular biomarkers, including blood pressure, endothelial function, and systemic inflammatory status (110).

Adipokine modulation may also contribute to these effects. Adiponectin exerts anti-atherogenic actions and regulates lipid metabolism and endothelial function through AMPK activation and reduction of vascular inflammation (93, 111).

Overall, polyphenols exert cardioprotective effects through integrated regulation of lipid metabolism, endothelial function, and redox homeostasis, thereby reducing the cardiometabolic risk associated with obesity.

### 3.5 Interaction with the gut microbiota

The gut microbiota is a key regulator of metabolic health and energy homeostasis, and its dysregulation is associated with obesity, metabolic syndrome, and insulin resistance (112). It influences host metabolism through nutrient processing, production of bioactive metabolites, and interactions with the immune system.

In particular, fermentation of dietary fibers leads to the production of short-chain fatty acids (SCFAs), such as acetate, propionate, and butyrate, which play a key role in regulating energy homeostasis, insulin sensitivity, and immune function (113).

SCFAs activate G-protein-coupled receptors, including GPR41 and GPR43, and modulate metabolic and inflammatory pathways. They also influence intestinal hormone secretion, including glucagon-like peptide-1 (GLP-1) and peptide YY (PYY), thereby improving insulin sensitivity and reducing systemic inflammation (112).

Butyrate also serves as a primary energy source for colonocytes and supports intestinal barrier integrity (113, 114).

In obesity, dysbiosis is associated with increased intestinal permeability and translocation of bacterial endotoxins, particularly lipopolysaccharides (LPS), into the systemic circulation. This phenomenon, known as metabolic endotoxemia, activates innate immune receptors such as TLR4 and contributes to systemic inflammation and insulin resistance (115).

Polyphenols interact bidirectionally with the gut microbiota, forming a dynamic diet–microbiota–host axis. Microorganisms metabolize polyphenols into low-molecular-weight derivatives that are often more bioavailable and biologically active. Conversely, polyphenols modulate microbiota composition by promoting the growth of beneficial species such as Bifidobacterium and Lactobacillus, thereby contributing to the restoration of eubiosis (116–118). Some polyphenols have been shown to increase the abundance of metabolically beneficial bacteria, including Akkermansia muciniphila, a species involved in maintaining intestinal barrier integrity and regulating glucose and lipid metabolism (119). Polyphenols may also increase SCFA production and shift microbiota composition toward a healthier metabolic profile (117, 118).

Another important mechanism involves modulation of bile acid metabolism. The gut microbiota converts primary bile acids into secondary bile acids, which act as ligands for receptors such as FXR and TGR5, involved in the regulation of lipid and glucose metabolism and inflammation. Polyphenols can influence this axis by modulating microbial composition and, indirectly, bile acid signaling (120, 121).

Consequently the microbiota–polyphenol axis represents a promising target for nutritional strategies against obesity-related cardiometabolic alterations, including in pediatric populations.

**Figure 1:**
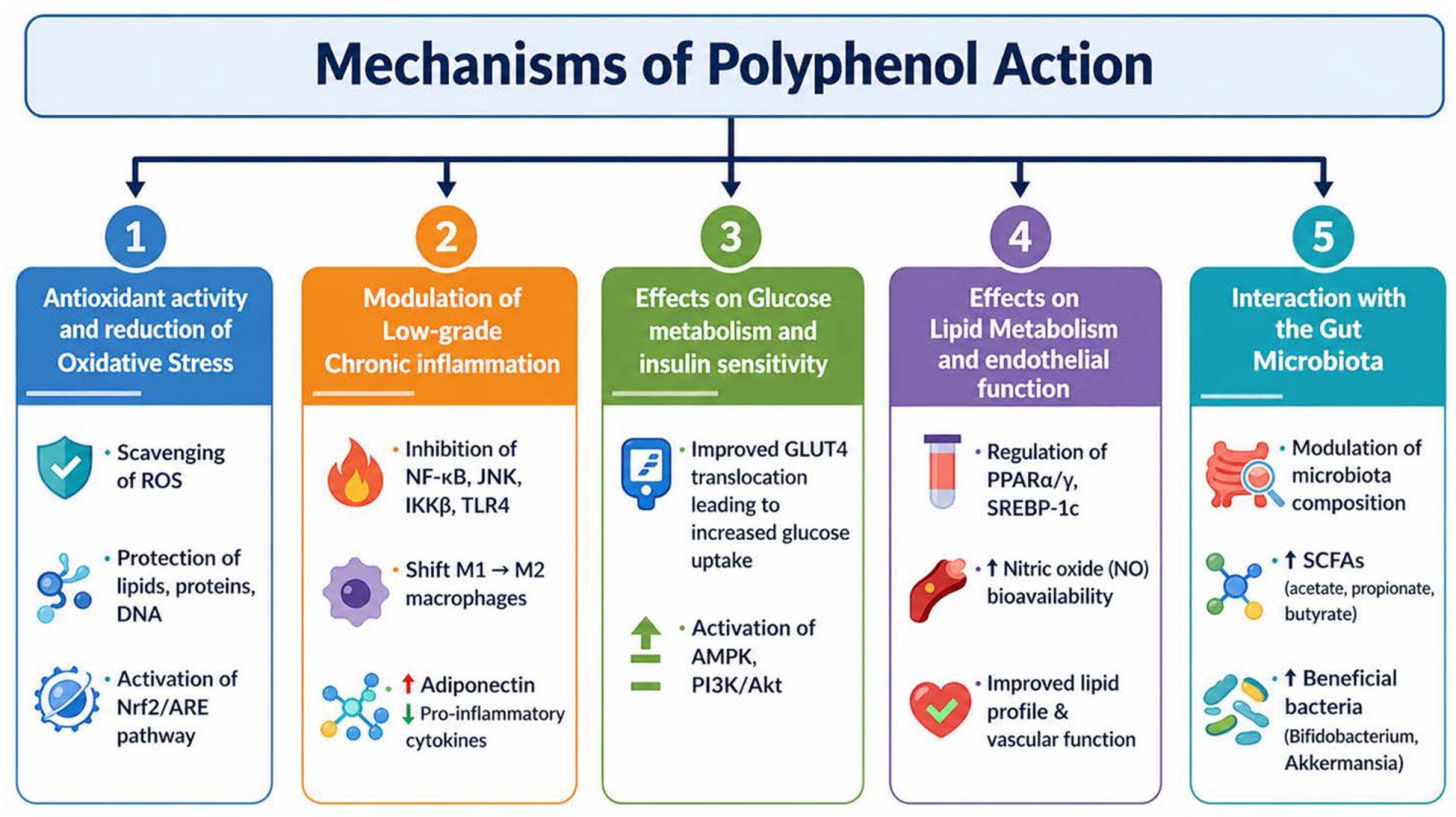
*Main biological mechanisms by which polyphenols may mitigate obesity-related cardiometabolic complications*. Polyphenols may act through complementary pathways, including antioxidant activity and reduction of oxidative stress, modulation of low-grade chronic inflammation, improvement of glucose metabolism and insulin sensitivity, regulation of lipid metabolism and endothelial function, and interaction with the gut microbiota.

In Figure 1, the main biological mechanisms through which polyphenols may counteract obesity-related cardiometabolic complications are schematized.

## 4 Clinical evidence in the pediatric population

### 4.1 Observational studies on polyphenol-rich diets and childhood obesity

The investigation of the relationship between polyphenol-rich dietary patterns and childhood obesity remains relatively limited compared with adult populations (122–125). Nevertheless, over the past decade, a growing number of cross-sectional and prospective cohort studies have begun to address this gap, primarily by evaluating overall diet quality or adherence to plant-based dietary patterns rather than directly quantifying polyphenol intake (34, 35, 126). This reliance on dietary patterns as proxies for polyphenol exposure represents a key methodological constraint that should be considered when interpreting the available evidence.

Several studies have examined adherence to the MD as a proxy for polyphenol-rich dietary patterns in pediatric populations. Higher adherence to the MD, typically assessed using indices such as the KIDMED score (127), has been consistently associated with lower BMI, reduced prevalence of overweight and obesity, and improved body composition parameters in children and adolescents across European cohorts (126, 128). One of the most influential contributions to this field is the IDEFICS (Identification and prevention of Dietary- and lifestyle-induced health EFfects In Children and infantS) study, a multicenter prospective cohort including 16,220 children aged 2–9 years from eight European countries (126). In this cohort, higher adherence to a Mediterranean-like dietary pattern was inversely associated with overweight, obesity, and percent fat mass, independently of age, sex, socioeconomic status, and physical activity (126). Prospective analyses further showed that higher baseline adherence was associated with a reduced risk of increases in BMI, waist circumference, and waist-to-height ratio over 2 years of follow-up (126). Similar findings were reported in the Growing Up Today Study II, a cohort of 10,918 US adolescents aged 8–15 years, in which higher adherence to the MD was associated with lower gains in BMI over both 3- and 7-year follow-up periods (129).

Consistent evidence also emerges from studies examining broader dietary patterns characterized by high consumption of plant-based foods. Higher intakes of fruits and vegetables have been associated with lower adiposity markers in pediatric populations, including lower BMI z-scores and reduced central adiposity (126). In addition, a large cross-sectional study conducted in Greece among 5,188 pre-school children aged 2–5 years reported that greater adherence to the MD was independently associated with a lower prevalence of overweight and obesity (130). A recent systematic review and meta-analysis further confirmed that higher adherence to healthy dietary patterns, including the MD, is associated with lower BMI and waist circumference in children and adolescents (131).

However, it is important to note that most of these studies do not directly quantify polyphenol intake, and the observed associations rely on dietary patterns used as proxies for polyphenol exposure. Studies directly assessing polyphenol intake in pediatric populations remain scarce and are generally limited in design. Among these, data from the HELENA study (34) indicated that adolescents with lower BMI tended to report higher polyphenol intake, although the cross-sectional nature of the analysis precludes causal inference. In addition, recent evidence using biomarkers of exposure suggests that increases in urinary total polyphenol excretion are associated with improvements in metabolic syndrome status in adolescents (132). While promising, these findings remain limited and require confirmation in larger and longitudinal studies.

Several methodological limitations further complicate the interpretation of the available evidence. Most observational studies rely on self-reported dietary assessment methods, such as food frequency questionnaires or 24-h recalls, which are subject to recall bias and measurement error, potentially leading to misclassification of polyphenol intake. Moreover, the use of dietary patterns as proxies does not allow disentangling the specific contribution of polyphenols from other components of a healthy diet. Residual confounding also remains a major concern, as higher consumption of polyphenol-rich foods is often associated with other health-promoting behaviors, including higher physical activity levels, better socioeconomic conditions, and overall healthier lifestyles. Overall, current observational evidence suggests that dietary patterns rich in plant-based foods, and therefore in polyphenols, are associated with more favorable weight status in children and adolescents. However, this evidence should be considered suggestive rather than conclusive, given the reliance on indirect measures of polyphenol exposure and the inherent limitations of observational study designs.

### 4.2 Evidence on insulin resistance and diabetes risk

Evidence on the role of dietary polyphenols in modulating insulin resistance and type 2 diabetes risk in pediatric populations remains limited, with few dedicated randomized controlled trials and most data deriving from observational studies or polyphenol-rich dietary patterns, particularly the Mediterranean diet (1, 34, 132, 133).

In the only double-blind RCT specifically testing isolated polyphenols in obese children, 62 girls aged 6–10 years received 400 mg/day decaffeinated green tea polyphenols (DGTP) for 12 weeks; the intervention produced no significant adverse effects and a modest trend toward reduced body-fat percentage, yet failed to demonstrate a statistically significant improvement in Homeostatic Model Assessment for Insulin Resistance (HOMA-IR) or fasting insulin levels (134, 135).

Notably, the available pediatric evidence is not designed to compare different metabolic phenotypes. The only randomized controlled trial specifically investigating isolated polyphenols was conducted in a high-risk subgroup, namely girls with obesity. In contrast, most observational studies have been performed in general pediatric populations, particularly adolescents. Therefore, current evidence does not allow direct comparisons between normal-weight and overweight/obese children, nor does it clarify whether the observed effects are consistent across different metabolic profiles.

Cross-sectional analyses from the large European HELENA cohort (approximately 2,000 adolescents aged 12–17 years) reported only modest inverse associations between total polyphenol and flavonoid intakes and BMI z-score, with no consistent relationship to metabolic syndrome (MetS) components or direct IR markers (e.g., HOMA-IR) after full adjustment for confounders (34).

Higher adherence to the MD has been linked in multiple cross-sectional studies to lower odds of IR in schoolchildren and adolescents; for example, poor adherence increased the likelihood of elevated HOMA-IR [odds ratio (OR) 1.31, 95% confidence interval (CI) 1.05–1.64] in Greek schoolchildren (136–138).

A recent systematic review and meta-analysis of 9 RCTs (pooled *n* = 577 participants ≤ 18 years) evaluating Mediterranean-diet interventions found no significant effects on glucose homeostasis, insulin, or HOMA-IR despite clear benefits on other cardiometabolic markers (1, 139).

Additional observational data from Spanish adolescents (*n* = 944, 11–14 years) and urinary total polyphenol excretion studies suggest indirect benefits on glucose metabolism *via* reduced inflammation and improved body composition, but direct causal evidence for polyphenols specifically lowering IR or diabetes risk in children remains sparse (132, 133, 140, 141).

### 4.3 Evidence on lipid profile and blood pressure

Clinical and observational evidence indicates modest but consistent favorable effects of dietary polyphenols and polyphenol-rich patterns on lipid profiles and blood pressure (BP) in pediatric populations, derived mainly from Mediterranean-diet interventions and biomarker-based studies (1, 142–144).

In cross-sectional analyses of Spanish adolescents, higher dietary polyphenol intake (especially flavonoids, phenolic acids, and stilbenes) was associated with improved serum lipids (HDL-C increase, TG and TC decrease) and better BP parameters after confounder adjustment (133, 145).

Urinary total polyphenol excretion (TPE) in large adolescent cohorts (*n* = 1151–1194, mean age ~12 years) showed negative associations with TG, TC, LDL-C in boys, and with systolic/diastolic blood pressure in girls (140, 141, 146).

The 2024 systematic review and meta-analysis of 9 RCTs (pooled *n* = 577 children and adolescents ≤ 18 years) demonstrated that Mediterranean-diet interventions produced statistically significant reductions in SBP (−4.75 mmHg), TG (−16.42 mg/dL), TC (−9.06 mg/dL), and LDL-C (−10.48 mg/dL), together with an increase in HDL-C (+2.24 mg/dL) (139). Individual RCTs included in the meta-analysis (16, 147–149) confirmed improvements in TG, TC/LDL-C, and SBP after 8–40 weeks of Mediterranean-diet promotion.

Higher urinary polyphenol levels have also been linked to better ideal cardiovascular health (CVH) scores, including optimal TC and BP categories, with gender-specific differences (145, 146, 150).

These sex-specific differences may be explained by several factors, including hormonal influences, differences in body fat distribution, and sex-related variability in metabolic and inflammatory responses (4, 8). In addition, variations in dietary patterns and gut microbiota composition between males and females may further contribute to differential responses to polyphenol intake. However, current evidence remains limited, and these hypotheses require further investigation in pediatric populations.

While effects are generally modest and more pronounced in higher-risk subgroups or when using objective biomarkers, study heterogeneity in polyphenol sources, doses, and durations limits definitive causal claims (144, 150, 151).

The main studies on dietary polyphenols and polyphenol-rich dietary patterns in pediatric populations are summarized in Table 2.

**Table 2:** Main clinical studies on dietary polyphenols or polyphenol-rich dietary patterns in pediatric populations^*^, with a focus on insulin resistance, lipid profile, blood pressure, and related cardiometabolic outcomes.

Reference	Study type	Population (n, age range)	Population type	Exposure/Intervention	Main outcomes	Key findings	Limitations
López-Gil et al. (16)	Systematic review and meta-analysis of 9 RCTs	577 children and adolescents (3–18 years)	Mixed pediatric population	MedDiet interventions (polyphenol-rich proxy)	SBP, DBP, TG, TC, HDL-C, LDL-C, glucose, HOMA-IR	↓ SBP, TG, TC, LDL-C; ↑ HDL-C; ⇄ glucose and HOMA-IR	Heterogeneity in intervention duration and adherence
Wisnuwardani et al. (34)	Cross-sectional (HELENA cohort)	657 European adolescents (12–17 years)	General population	Total polyphenols & flavonoid intake	MetS components, BMI z-score, IR markers	⇄MetS/IR; ↓BMI (modest);	Self-reported intake, low MetS prevalence
Laveriano-Santos et al. (133)	Cross-sectional	944 adolescents (11–14 years)	General population	Dietary polyphenol intake (quintiles)	WC, BP, HDL-C, glucose, TG	↓ WC, BP, TG; ↑ HDL-C	Cross-sectional design
Xie et al. (134)	RCT, double-blind, placebo-controlled	62 obese girls (6–10 years)	Obese children	400 mg/d DGTP vs placebo, 12 weeks	Body fat %, precocious puberty markers, HOMA-IR, safety	↓ body fat (trend);⇄ HOMA-IR	Small sample, short duration
Die Yao et al. (135)	Safety analysis/follow-up of the same RCT	62 obese girls (6–10 years)	Obese children	400 mg/d DGTP, 12 weeks	Safety and adverse events	No adverse health effects observed	Not powered for efficacy endpoints (safety-focused)
George et al. (136)	Cross-sectional	Schoolchildren (9–13 years)	General population	MedDiet adherence	IR, MetS components	↑ IR risk with low adherence	Self-reported diet
Mohammadi et al. (137)	Cross-sectional	203 adolescents (12–18 years)	Overweight/obese	MedDiet adherence	MetS, HOMA-IR	↓ IR, MetS with higher adherence	Observational
Pavlidou et al. (138)	Cross-sectional	Preschool children (3–6 years)	General population	MedDiet adherence	Obesity, cardiometabolic risk	↓ obesity risk	Self-reported
Dubey et al. (139)	Systematic review and meta-analysis	Children and adolescents ( ≤ 18 years)	Mixed pediatric population	MedDiet adherence	MetS risk	↓ MetS risk with adherence	Heterogeneity across studies
Arancibia-Riveros et al. (132)	Longitudinal cohort	Adolescents (~12–17 years)	General population	Urinary TPE	MetS reversal, inflammation	↑ MetS reversal	Observational
Laveriano-Santos et al. (140)	Cross-sectional	1,194 Spanish adolescents (~12 years)	General population	Urinary total polyphenol excretion (TPE)	Lipids, BP, body fat	↓ TG, TC, LDL-C (boys); ↓ BP (girls)	Observational
Laveriano-Santos et al. (141)	Cross-sectional	1,151 adolescents (mean 12.04 years)	General population	Urinary TPE	Ideal CVH metrics, lipids, BP	↑ CVH score; ↓ lipids, BP	Cross-sectional, gender differences
Godos et al. (142)	Systematic review and meta-analysis	Observational studies (incl. youth)	Mixed pediatric population	Dietary polyphenols	BP, hypertension	↓ BP	Mixed ages
Laveriano-Santos et al. (145)	Cross-sectional	560 adolescents (mean 12.02 years)	General population	Urinary microbial phenolic metabolites	MetS features, WC, BP, and glucose	↓ WC, BP, and glucose	Observational
Ramírez-Garza et al. (146)	Cross-sectional	Adolescents (~12 years)	General population	Urinary TPE + fruit/vegetable intake	BP	↓ BP	Observational
Muros et al. (148)	RCT	84 children (8–12 years)	Mixed pediatric population	MedDiet + EVOO 8 weeks	Lipids, SBP	↓ TG, TC; ↑ HDL-C; ↓ SBP	Short duration
Fernandez-Ruiz et al. (147)	RCT	84 overweight/obese children (8–12 years)	Overweight/obese	MedDiet program 40 weeks	Lipids, SBP	↓ TC, LDL-C; ↓ SBP	Variable adherence
Velázquez-López et al. (149)	RCT	61 obese youth (6–18 years)	Obese children	MedDiet 12 weeks	Lipids, MetS	↓ TG, TC, LDL-C	Small sample
Buckland et al. (150)	Prospective observational	Adolescents followed to young adulthood	General population	Mediterranean-style diet score	CMR profile at 24 years	↑ adult cardiometabolic profile	Long follow-up but indirect polyphenol focus
Massini et al. (151)	Observational	Children with dyslipidemia ( ≤ 18 years)	High-risk population	Updated KIDMED (MedDiet) score	Lipid profiles	↓ lipids	Cross-sectional
Bruna-Mejias et al. (152)	Systematic review	Children and adolescents with MetS ( ≤ 18 years)	MetS population	MedDiet vs. other diets	BMI, WC, TG, glucose, HOMA-IR	↓ BMI, WC, TG, glucose, HOMA-IR	Heterogeneity
Kafyra et al. (153)	Cross-sectional	766 Greek + 287 French teenagers (13–17 years)	General population	Dietary patterns (incl. polyphenol-rich)	BP, lipids	Western diet → ↑ BP, lipids	Cross-sectional

↓ decrease; ↑ increase; ⇄ no significant change; BP, blood pressure; CMR, cardiometabolic risk; CVH, cardiovascular health; DGTP, decaffeinated green tea polyphenols; EVOO, extra virgin olive oil; HDL-C, high-density lipoprotein cholesterol; HOMA-IR, Homeostatic Model Assessment for Insulin Resistance; IR, insulin resistance; LDL-C, low-density lipoprotein cholesterol; MedDiet, Mediterranean diet; MetS, metabolic syndrome; NO, nitric oxide; SBP, systolic blood pressure; DBP, diastolic blood pressure; TC, total cholesterol; TG, triglycerides; TPE, urinary total polyphenol excretion; WC, waist circumference. *Most studies were conducted in general pediatric populations or in children/adolescents with overweight or obesity; no pediatric studies specifically evaluating polyphenol intake in type 2 diabetes were identified.

## 5 Discussion

The available evidence supports the biological plausibility of dietary polyphenols as modulators of several pathways involved in pediatric obesity and cardiometabolic risk, including oxidative stress, chronic low-grade inflammation, insulin resistance, dyslipidemia, endothelial dysfunction, and gut microbiota imbalance (13, 59, 77, 79, 103). Overall, observational findings and the still limited pediatric clinical data suggest that polyphenol-rich dietary patterns may contribute to a more favorable metabolic profile in children and adolescents with obesity (146–153). In particular, Mediterranean-style dietary patterns appear especially relevant, given their high content of plant-derived bioactive compounds and their association with more favorable adiposity and cardiometabolic outcomes in pediatric populations (146–153).

A central point of discussion concerns the different interpretative approaches within the field. One perspective emphasizes that the potential health effects of polyphenols should be understood within the broader context of whole dietary patterns, particularly those rich in fruits, vegetables, legumes, whole grains, nuts, and other minimally processed plant foods (46–58, 147**?** –153). According to this view, polyphenols likely act in synergy with fiber, vitamins, minerals, and other food-matrix components, making food-based approaches more clinically meaningful than isolated supplementation. A more reductionist perspective, by contrast, focuses on specific polyphenol classes or single compounds and attempts to identify direct metabolic effects and dose-response relationships through targeted interventions or supplementation studies (59, 73–76, 86–90, 98–108). This tension underlies an important controversy in the field: whether the observed cardiometabolic benefits are attributable to polyphenols *per se* or instead reflect the overall quality of polyphenol-rich diets and associated healthy lifestyles.

This controversy is closely linked to several fundamental conceptual and methodological issues. Polyphenol exposure is intrinsically complex, as biological effects depend not only on intake amount but also on food matrix, bioavailability, metabolism, and interindividual differences in gut microbiota composition (19–21, 24–31). In addition, pediatric obesity is a highly heterogeneous condition influenced by age, sex, pubertal stage, adiposity severity, genetic background, socioeconomic environment, and lifestyle behaviors, all of which may modify responses to dietary interventions (4, 8–12, 19–21). These factors complicate causal interpretation and make it difficult to disentangle the specific contribution of polyphenols from the broader effects of diet quality and lifestyle. A further major issue is methodological: most available studies are observational, cross-sectional, or short-term, and polyphenol intake is often estimated through self-reported dietary questionnaires or recalls linked to food-composition databases rather than through validated biomarkers of intake and metabolism, increasing the risk of residual confounding, measurement error, and exposure misclassification.

In addition to the cardiometabolic parameters discussed, other biomarkers such as inflammatory markers (e.g., C-reactive protein, interleukin-6, TNF-α) and indices of glucose metabolism (fasting glucose, insulin, HbA1c) are recognized components of cardiometabolic risk. However, existing studies are few, often involve small sample sizes, and frequently report these biomarkers as secondary outcomes, with inconsistent findings (139). For this reason, they were not specifically addressed as primary endpoints in the present review.

A further limitation of the current literature is the marked heterogeneity of study populations and the lack of subgroup-specific evidence. Available studies include both general pediatric cohorts and children or adolescents with overweight or obesity, but they are rarely designed to compare these groups directly. Moreover, high-risk phenotypes, such as pediatric type 2 diabetes mellitus, remain largely underrepresented. As a result, it is still unclear whether the potential cardiometabolic benefits of polyphenol-rich diets are consistent across normal-weight, overweight/obese, and diabetic children. This limitation is clinically relevant, as baseline metabolic status may influence both the magnitude and the mechanisms of response to dietary polyphenols.

Current research gaps remain substantial. High-quality pediatric data are scarce, especially in children younger than 10 years, and much of the current interpretation still relies on adolescent cohorts, adult studies, or experimental models. Pediatric randomized controlled trials are limited in number and are generally characterized by small sample sizes, short intervention periods, heterogeneous exposures, and a predominant focus on surrogate markers rather than clinically relevant long-term endpoints, such as sustained improvements in insulin resistance or reduced incidence of type 2 diabetes. Safety data are also insufficient, particularly with regard to high-dose polyphenol supplementation in children, for which long-term tolerability and possible interactions remain inadequately explored. Additional gaps concern ethnic and genetic diversity, *in vivo* gut microbiota–polyphenol interactions, longitudinal cardiovascular outcomes, and the possibility of tailoring interventions according to baseline diet, metabolic phenotype, or genetic background.

From a clinical perspective, current evidence is not sufficient to support specific recommendations for isolated polyphenol supplementation in pediatric populations. At present, the most evidence-based implication is the promotion of healthy dietary patterns naturally rich in polyphenols within comprehensive lifestyle interventions. In pediatric practice, these findings are best translated into food-based strategies aimed at improving overall diet quality rather than increasing the intake of individual compounds. In this context, Mediterranean-style dietary patterns are particularly relevant because of their consistent association with more favorable cardiometabolic profiles in children and adolescents (147–153), and because they are coherent with current family-based lifestyle approaches to pediatric obesity management.

Potential developments in the field will depend on more rigorous and clinically relevant pediatric research. Future studies should prioritize larger, longer-duration, pediatric-specific randomized controlled trials with standardized assessment of polyphenol exposure, preferably using validated biomarkers, and with clinically meaningful cardiometabolic outcomes. Mechanistic investigations integrating microbiome profiling, metabolomics, inflammatory markers, and vascular endpoints will be important for clarifying the pathways through which polyphenols may influence early-life metabolic health. In particular, deeper investigation of microbiota-related mechanisms may help explain interindividual variability in response and better define the preventive role of polyphenol-rich diets in childhood and adolescence. More broadly, the field may progressively move toward precision nutrition approaches aimed at identifying which children are most likely to benefit according to baseline dietary habits, microbiota composition, metabolic phenotype, or genetic background.

In conclusion, dietary polyphenols represent a promising but still evolving area of research in pediatric obesity and cardiometabolic prevention. Their relevance is supported by coherent mechanistic evidence and by encouraging observational and early clinical findings, but their direct clinical application remains limited by conceptual controversy, methodological weaknesses, and insufficient pediatric-specific evidence. For now, the most appropriate and evidence-based approach is to encourage adherence to healthy dietary patterns naturally rich in polyphenols as part of broader lifestyle interventions, while further pediatric studies clarify the magnitude, mechanisms, safety, and long-term clinical relevance of these effects.
